# Processing and Thermal Conductivity of Bulk Nanocrystalline Aluminum Nitride

**DOI:** 10.3390/ma14195565

**Published:** 2021-09-25

**Authors:** Matthew A. Duarte, Vivek Mishra, Chris Dames, Yasuhiro Kodera, Javier E. Garay

**Affiliations:** 1 Materials Science and Engineering Program, Mechanical and Aerospace Engineering Department, University of California, San Diego, CA 92093, USA; m8duarte@eng.ucsd.edu; 2 Department of Mechanical Engineering, University of California, Berkeley, CA 94720, USA; vivek.mishra@berkeley.edu (V.M.); cdames@berkeley.edu (C.D.)

**Keywords:** aluminum nitride, nano powder, nanocrystalline, reduction/nitridation, using current activated pressure assisted densification, CAPAD, SPS, high thermal conductivity, 3ω

## Abstract

Producing bulk AlN with grain sizes in the nano regime and measuring its thermal conductivity is an important milestone in the development of materials for high energy optical applications. We present the synthesis and subsequent densification of nano-AlN powder to produce bulk nanocrystalline AlN. The nanopowder is synthesized by converting transition alumina (δ-Al_2_O_3_) with <40 nm grain size to AlN using a carbon free reduction/nitridation process. We consolidated the nano-AlN powder using current activated pressure assisted densification (CAPAD) and achieved a relative density of 98% at 1300 °C with average grain size, $\bar{d}$~125 nm. By contrast, high quality commercially available AlN powder yields densities ~75% under the same CAPAD conditions. We used the 3-ω method to measure the thermal conductivity, *κ* of two nanocrystalline samples, 91% dense,$\;\bar{d}$ = 110 nm and 99% dense, $\bar{d}$ = 220 nm, respectively. The dense sample with 220 nm grains has a measured *κ* = 43 W/(m·K) at room temperature, which is relatively high for a nanocrystalline ceramic, but still low compared to single crystal and large grain sized polycrystalline AlN which can exceed 300 W/(m·K). The reduction in *κ* in both samples is understood as a combination of grain boundary scattering and porosity effects. We believe that these are finest $\bar{d}$ reported in bulk dense AlN and is the first report of thermal conductivity for AlN with ≤220 nm grain size. The obtained *κ* values are higher than the vast majority of conventional optical materials, demonstrating the advantage of AlN for high-energy optical applications.

## 1. Introduction

Thermal management is a critical task for high energy optical systems. A key strategy in thermal management is developing transparent high thermal conductivity materials and aluminum nitride (AlN) is a promising candidate. As a wide band gap semiconductor, AlN has a wealth of current and possible opto-electronic applications in light emission including display technology, lighting and lasing [1,2,3,4,5]. AlN is an important advanced material in technology today due to many noteworthy properties such as excellent dielectric properties, low coefficient of thermal expansion, nontoxicity, and chemical/high temperature stability especially in non-oxidizing environments. Recently there has been renewed interest in AlN for thermal management applications such as substrates/heat sinks [6] and fillers for adhesives [7]. Arguably AlN’s most distinctive characteristic is its high thermal conductivity, *κ*, which is one of the highest amongst non-metallic materials and is in fact, higher than many metals [8]. With significant effort in materials processing, pure polycrystalline AlN has been demonstrated with *κ* = 272 W/(m·K) [9] which is 95% of the measured single crystal [10]. AlN’s high thermal conductivity stems from high phonon group velocity and long phonon mean free paths. Slack estimated a theoretical room-temperature thermal conductivity for single crystal AlN, *κ* = 319 W/(m·K) [10] by extrapolating measured single crystal conductivities to an oxygen free value. Oxygen impurities in AlN are considered intrinsic since they are virtually unavoidable and like other point defects, they can lower *κ* by scattering phonons and decreasing mean free paths. Similarly, grain boundaries can play a role in thermal conductivity [11]. We have previously shown a grain size dependence of *κ* in rare earth doped AlN with average grain sizes, $\bar{d}$ in the 2–4 μm range [12]. We are not aware, however, of studies of *κ* in AlN with grain sizes in the nanocrystalline regime.

The same high temperature stability that makes AlN attractive also renders it notoriously difficult to densify from powders [13]. For example, pressure-less sintering of micrometer sized AlN powder at temperatures as high as 1900 °C does not result in complete densification [14]. Therefore, developing sintering additives became an important component of research related to synthesis and processing of AlN [14,15]. Using sub-micrometer sized powder, sintering additives and pressure have lowered the required densification temperature to 1450 °C resulting in polycrystalline AlN with sub-micrometer grains [16].

Nanocrystalline grain sizes are important both for reducing powder densification temperatures and for tailoring the properties of the resultant polycrystalline materials. There are some examples of the success in the production of nano AlN powder and the densification to obtain full density with controlled grain growth. Gas phase nitridation is a proven approach to synthesize nano-size AlN powder [17,18,19,20,21]. Also, researchers used current activated pressure assisted densification (CAPAD) to densify nano-AlN powder and the final grain size was around 200 nm in nearly fully dense ceramics [22,23]. In turn, nanocrystalline ceramics often have improved mechanical properties but significantly reduced thermal conductivity [11]. In order to achieve high transparency, it is critical to have fine grain sizes for AlN. Optically anisotropic crystal structures like AlN can have significant scattering in randomly oriented polycrystalline form; grain sizes smaller than the wavelength of light reduce scattering losses [24].

For those reasons, producing AlN bulk materials with nano grain size and measuring its thermal conductivity is an important for the development of innovative material for high energy optical application. In this paper we present a method for the synthesis of nano-AlN powder by an alumina (Al_2_O_3_) to AlN conversion route using a carbon free ammonia gas nitridation process. In order to ensure fine grain sizes, we chose transition alumina (δ-Al_2_O_3_) with about 40 nm grain sizes and used a relatively low nitridation temperature. We densified the nano-AlN powder using CAPAD and found that the nano-AlN powder achieves significantly higher densities than high quality commercially available powder at the same processing conditions. We also report the thermal conductivity of two nanocrystalline samples with different relative density (porosity) and $\bar{d}$. 

## 2. Materials and Methods

### 2.1. Powder Processing

All samples were processed from commercially available transition-aluminum oxide (measured as δ-Al_2_O_3_, 99.99% purity) powder, <40 nm particle size, BET specific surface area >150 m^2^/g Inframat Advanced Materials, using a tube furnace with flowing ammonia gas, NH_3_ (anhydrous grade 99.99% Purity). The powder was placed onto an alumina crucible directly in the center of the tube furnace. Before flowing NH_3_ gas, the tube itself was evacuated by a mechanical vacuum pump. The tube furnace was brought to a temperature of 200 °C and held for 20 min to remove absorbed moisture. After the initial hold time, NH_3_ gas was then introduced at a rate of 500 mL/min. The temperature was then raised and held at temperatures of 1100–1400 °C, at a heating rate of 5 °C/min. The hold time of the maximum temperature was 1–24 h. The temperature was then cooled to 50 °C at a rate of 5 °C/min. We tried to minimize the exposure to the atmosphere when transferring the sample to a glove box filled with Ar for subsequent processing.

### 2.2. Consolidation of AlN Powder

For consolidation using CAPAD, 0.25 g of powder were loaded into a graphite die and plunger set under an argon atmosphere in an inert atmosphere glovebox. The die and plunger assembly were then loaded into our custom-built CAPAD apparatus [25]. Once the sample was in the CAPAD, the chamber was evacuated to 3 × 10^−2^ Torr. Densification was conducted using 105 MPa of applied pressure and a heating rate of approximately 300 °C min^−1^. The hold temperatures ranged from 1100–1600 °C, and the hold times were approximately 10 min. Temperature was measured using an optical pyrometer focused on the die wall.

### 2.3. Structural Characterization

A XL30-FEG Scanning Electron Microscopy (SEM) (FEI Thermo Fisher Scientific, Waltham, MA, USA) with accelerating voltages ranging from 10–20 kV was used to characterize morphology of powder and microstructure of bulk sample. Working distances in the system were approximately 5 mm. X-ray Diffraction patterns were attained using a X’Pert X-ray Diffraction (XRD) (Malvern Panalytical, MA, USA) system equipped with a copper source/proportional counter, with a step size of 0.01313 degrees and 0.30 s per step. Samples were pressed flat on a zero-background plate and measured with no stage rotation. A maximum slit opening was used for both the divergence slit and anti-scatter slit in the incident beam. A tension of 45 kV and a current of 40 mA was used for all measurements. 

The relative density of the samples was measured using the Archimedes method using 3.26 g/cm^3^ as the AlN theoretical density. The average grain size was measured using image analysis of SEM images of powder and fracture surfaces of bulk sample. Averages were calculated from measurements of at least 100 grains; the measurement was continued until no change on the grain size histogram was observed. 

### 2.4. Thermal Conductivity Measurement

The electro-thermal 3ω method [26] was used to measure the thermal conductivity of the AlN samples from room temperature to 77 K. The sample were mounted inside a liquid nitrogen cooled open flow cryostat. The heater lines were patterned by e-beam evaporation of gold through a shadow mask and were nominally 1.5 mm long, 60 µm wide, and 100 nm thick. Vacuum compatible thermal grease (Apiezon) was used to enhance the thermal contact between the sample and the cryostat cold finger. For each cryostat temperature set point, the sample temperature was determined from the temperature dependent resistance *R*(*T*) calibration of the gold heater line. This *R*(*T*) function is expected to be highly linear from well above room temperature down to liquid nitrogen temperatures [27] and was determined experimentally by extrapolation from measurements taken in the vicinity of room temperature.

The standard 3ω slope method was used to calculate the thermal conductivity at each temperature point. This is a suitable analysis approach since: (i) both the grain sizes of the polycrystalline AlN and the typical heat carrying phonon mean free paths of AlN [28] are much smaller than the heater line half width, allowing the sample to be treated as a homogeneous effective medium in the continuum approximation; and (ii) the smallest estimated thermal diffusion lengths for the heater line frequencies used (50–100 Hz) are at least five times larger than the heater line half width [29].

## 3. Results and Discussion

### 3.1. Powder Processing

AlN powder is often commercially produced through a gas-solid based direct nitridation of metal aluminum. The direct nitridation of aluminum metal is highly exothermic reaction and usually requires a post-synthesis milling process which can introduce potential impurities. The nitridation of aluminum oxide, aluminum hydroxide and other compounds have also been shown. One well known approach, called the carbothermal reduction–nitridation process, uses mixtures of aluminum oxide powder + carbon and nitrogen or ammonia. Suehiro et al. successfully employed gas mixtures of NH_3_-CH_3_H_8_ for synthesizing AlN powder and maintained the original morphology and shape of starting alumina compounds. [17,18,19,20,21]. These studies showed that the use of carbon containing components reduces the nitridation temperature. However, it is well known that the carbothermal reduction–nitridation process method requires an additional post-oxidation step to remove the residual carbon [20,21]. We conjectured that carbon residue on the surface of AlN powder might inhibit densification and adversely affect its properties, so we chose a carbon-free nitridation (pure ammonia) environment.

The stability of Al_2_O_3_ is known to be related to the surface area and therefore size dependent [30]. The most stable form is α-Al_2_O_3_; the δ-Al_2_O_3_ and γ-Al_2_O_3_ occur at smaller particle sizes and can be thought of as metastable or transition phases. In order to obtain very fine grain size after nitridation we chose transition alumina (δ-Al_2_O_3_) with <40 nm grain size as a starting material. The reaction mechanism (nitridation) of alumina using ammonia to form AlN can be tracked using XRD. First, we changed the temperature to discover global phase change and determined optimum temperature. Second, we change the holding time and focused the detail of phase presence to determine optimize holding time at selected temperature.

**Figure 1:**
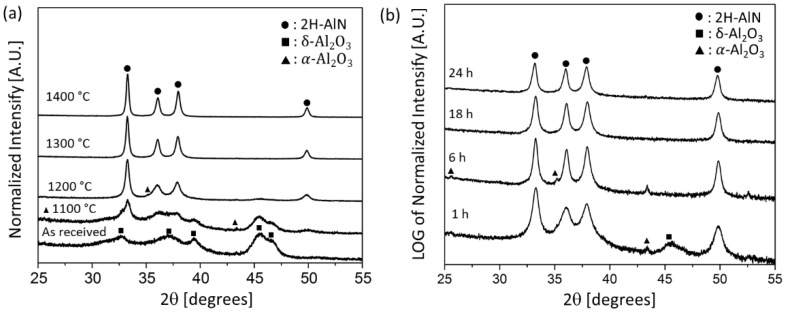
The effect of nitridation time and temperature on phase evolution of powders (**a**) XRD patterns of samples at various times. (**b**) XRD patterns of samples at 1200 °C for various times. Normalized intensity is plotted with in log scale to emphasize minor phases.

Figure 1a shows XRD patterns of nitrided samples produced using different processing temperatures held for 1 h at temperature along with the commercial transition alumina starting powder for reference. The starting powder shows broad low crystallinity peaks typical of δ-Al_2_O_3_. The sample processed at 1100 °C exhibits weak diffraction peaks corresponding to α-Al_2_O_3_ (closed triangle dots). The peak at 2θ = 33.2° grows and exhibits a relatively sharp peak top. Also, small humps at 36.0° and 49.8° are observed. These suggest partial conversion of α-Al_2_O_3_ from the original transition alumina. This is in line with previous observations that formation of α-Al_2_O_3_ generally takes place above 1000 °C [31]. By elevating the temperature to 1200 °C, there was a drastic change in diffraction peaks; four clear peaks were observed at 33.2°, 36.0°, 37.9°, and 49.5° corresponding to the 100, 002, 101, and 102 planes of 2H-AlN, respectively. The nitridation seems fully completed at 1300 °C with 1 h hold since the small hump near 46° corresponding to δ-Al_2_O_3_ is no longer present. The change of XRD pattern peaks by nitriding over 1300 °C was not significant. Figure 1b shows the effect of holding time on the nitridation at 1200 °C under NH_3_ gas flow. The normalized intensity on the vertical axis is plotted in log scale to emphasize the minor peaks. The sample held for 1 h shows a predominance of AlN and weak peaks of α-Al_2_O_3_ and δ-Al_2_O_3_. After holding for 18 h or longer, only AlN phase was observed. Performing the nitridation for longer than 18 h did not change the XRD pattern of products.

**Figure 2:**
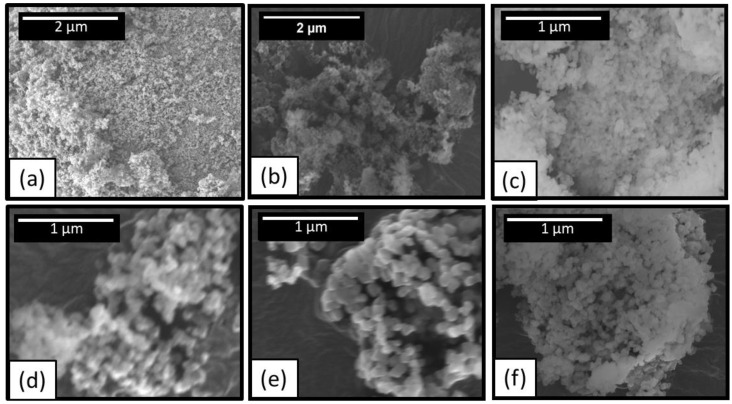
SEM micrographs of nanopowders. (**a**) unreacted δ-Al_2_O_3_. Samples nitrided at (**b**) 1100 °C, (**c**) 1200 °C (**d**) 1300 °C, and (**e**) 1400 °C for 1 h. (**f**) is 1200 °C for 18 h.

Figure 2 shows SEM micrographs of the powders nitrided at various conditions. These images reveal that increasing nitridation temperatures cause an increase in the grain size. The granular particle morphology without noticeable facet or crystal habit and phase shown in phase diagram of AlN-Al_2_O_3_ suggested that the growth mechanism of powder (mass transport from adjacent grains) during nitridation process is possibly surface and volume (solid state) diffusion.

**Figure 3:**
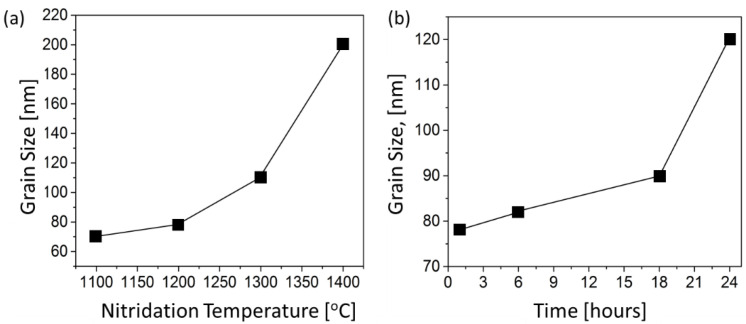
The effect of nitridation condition on the grain size of reacted powders. The grain size was measured from SEM. (**a**) Nitridation temperature vs. grain size for various temperature held for 1 h. (**b**) Holding time at 1200 °C vs. grain size.

The effect of temperatures on grain size is more clearly appreciated in Figure 3a which plots average grain size vs. nitridation temperature. At 1100 °C, the measured grain size was ~70 nm which increased to ~200 nm at 1400 °C. The $\bar{d}$ of samples processed at 1100 °C and 1200 °C are below 100 nm which is important for attaining dense nano-AlN.

In reactions involving thermal powder processing, grain coarsening can be caused by two mechanisms: (1) mass transfer in the form of sintering and (2) volume change by conversion from reactant to product (i.e., conversion of Al_2_O_3_ to AlN). In order to assess the dominant grain growth mechanism, we can calculate the change in volume expected for the conversion of Al_2_O_3_ to AlN. The molar volume of a particular phase, *i* is:

(1) $$V_{m,i}=\frac{N_{A}V_{cell}}{Z}$$

where *N_A_* is the Avogadro constant, *V_cell_* is the unit cell volume and *Z* is the number of formula unit in the unit cell. *V_m_*_Al2O3_ = 14.20 and *V_m_*_AlN_ = 12.58 cm^3^ per one mole of Al. All values were obtained from data base (ICSD99836: δ-Al_2_O_3_, ICSD54697:AlN) and normalized to be per one mole of Al. If we assume spherical grains, the effect of the molar volume change on the radii of converted AlN is

(2) $$r_{\mathrm{AlN}}=r_{\mathrm{Al}2O3}\sqrt[{3}]{\frac{V_{m,\mathrm{AlN}}}{V_{m,\mathrm{Al}2O3}}}$$

At 1100 °C, the measured grain size was 70 nm and the phase was mainly unreacted δ-Al_2_O_3_ (observed by XRD) which became AlN with 80 nm grain size at 1200 °C. Using Equation (2), one finds that *r*_AlN_ should have decreased to 67 nm when *r*_Al2O3_ was 70 nm if the mechanism were dominated by the molar volume change. Thus, it is clear that the change in grain size is caused by mass transfer such as sintering. As the temperature increases to 1300 and 1400 °C, there is a significant increase in grain size approximately around 110 and 200 nm, respectively.

The influence of holding time at 1200 °C on the grain size of sample is presented in Figure 3b. Grain growth in fully dense systems is expected to have a power law time dependence. For example, normal (diffusion controlled) grain growth kinetics can be described using ${\bar{d}}^{2}-d_{o}^{2}=kt$ where *d_o_* is the initial average grain size (at *t* = 0), k is the grain growth rate which is related to grain boundary mobility. Such behavior is not expected in our powder samples since of the large amounts of porosity in loosely packed powder. In addition, the XRD results confirm that the grain growth at 1200 °C is accompanied by Al_2_O_3_ to AlN conversion (holding times below 6 h still contains unreacted Al_2_O_3_ while samples held for 18 h and over shows only AlN). We chose powder nitrided at 1200 °C for 18 h (shown in Figure 2f) for further CAPAD processing due to the combination of full AlN conversion and small grain size (90 nm). Henceforth we refer to this powder as nano-AlN powder.

### 3.2. Densification of Nano-AlN Powder

**Figure 4:**
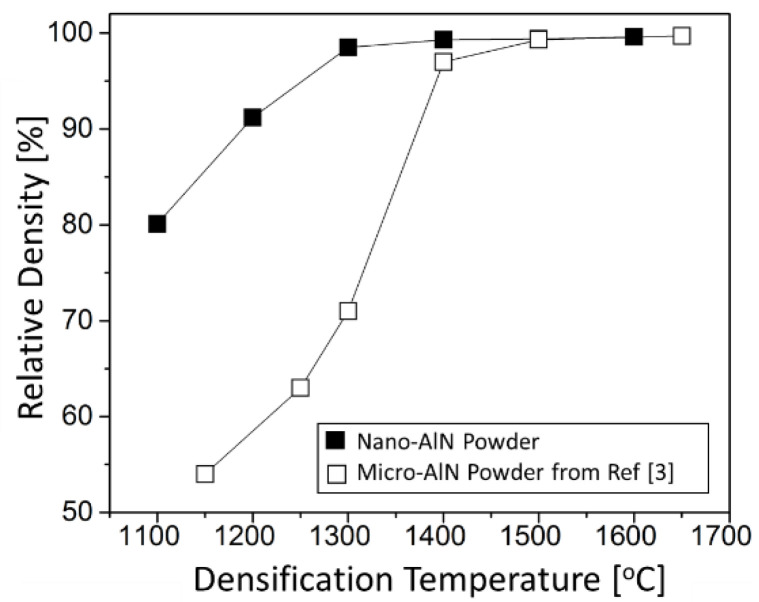
The effect of CAPAD temperature on relative density of samples densified from. Black square dots show the powder nitridated at 1200 °C for 18 h. Open square dots shows the commercial AlN powder [3].

Figure 4 shows the effect of CAPAD temperature on the relative density of samples produced from nano-AlN powder along with samples densified from micrometer sized commercial powder we reported previously [3] densified using similar pressures and processing times. As expected, the relative density of AlN densified from commercial powder increased with CAPAD temperature. The high translucency/transparency of the CAPAD processed samples from this commercially available powder is a testament to its high quality [3]. There is a sharp rise in density between 1300 °C where the density is 70% and 1400 °C where the sample is >95% dense. The AlN from commercial powder is fully dense at 1500 °C. These results confirm the advantages of CAPAD since as mentioned earlier, pressure-less sintering of additive-free micrometer sized powder at 1900 °C does not produce full density [14]. These results are in line with CAPAD densified sub-micron AlN powder that also showed full density at 1500 °C without additives [22]. On the other hand, the nano-AlN powder reaches over 80% relative density after the remarkably low temperature of 1100 °C. The density gradually increased with temperature and is over 97% at 1300 °C. This high density is noteworthy since AlN from commercial powder showed only around 70% density at the same temperature and pressure. The sample processed from nano-AlN powder at 1400 °C was fully dense. It is worth emphasizing that this temperature is lower than was required for full densification of AlN using sintering additives (1450 °C) [16]. We attribute this difference to the grain size of the powder, ~90 nm. Densification of nano-AlN powders (~100 nm) has been recently shown to reduce the required densification temperature in pressure-less sintering; full density was achieved at ~1850 °C without sintering additives [14,32,33].

**Figure 5:**
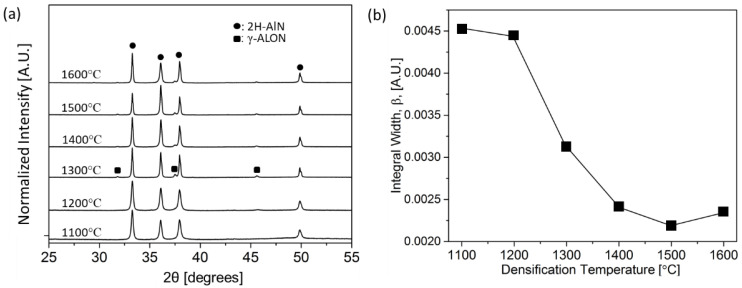
(**a**) XRD patterns of sample densified at different CAPAD temperatures. AlN powder was nitrided at 1200 °C for 18 h prior to the densification. (**b**) Integral width of (100) peak vs. CAPAD temperature.

Figure 5a shows XRD patterns of bulk AlN for varying CAPAD processing temperatures. All of the major peaks (at 33.2°, 36.0°, 37.9° and 49.8° corresponding to 100, 002, 101 and 102) belong to 2H-AlN, confirming that AlN is the dominant phase. Above 1300 °C, there are small peaks identified as gamma aluminum oxynitride (γ-ALON) which is nonstoichiometric Spinel structure (Fd-3m). γ-ALON peaks that were not detectable in the nano-powder. This suggests that there were oxygen impurities present in the nanopowder, likely on the surface of the powder, making it difficult to detect using XRD. The presence of oxygen impurities in AlN is not surprising since it is very difficult to prevent oxygen contamination in AlN. The peak shape of AlN changed slightly with temperature. The samples processed at higher temperature exhibited Kα_2_ peak splitting especially at higher angles suggesting the crystallinity of sample increased with temperature, which is consistent with grain size measurements to be discussed below. Figure 5b. shows the integral width measured from 100 peak of 2H-AlN. The change in integral width is minor between the temperature 1100 °C and 1200 °C and showed a sharp decrease at 1300 °C. An attempt was made to estimate the crystallite size using the Scherrer equation with Shape factor 0.9. The Kα2 contribution was removed and the instrumental broadening was not removed. The calculated sizes were 56, 58, 83, 107, 118, and 109 nm for the sample densified at 1100, 1200, 1300, 1400, 1500, and 1600 °C, respectively. The reason for the decrease of crystallite size at 1600 °C is unknown. These values were less accurate since the instrumental broadening was not removed which gives under estimated crystallite size.

**Figure 6:**
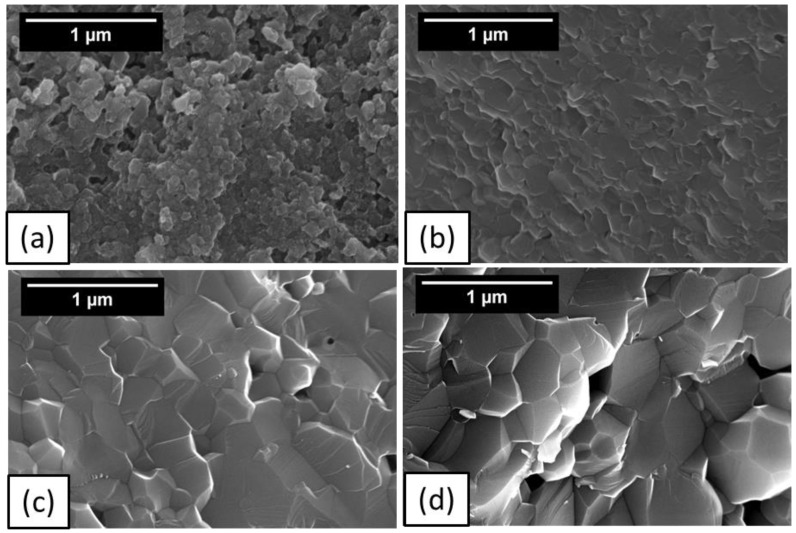
SEM micrographs of fractured surfaces of AlN bulk sample densified at (**a**) 1200 °C, (**b**) 1300 °C, (**c**) 1500 °C and (**d**) 1600 °C.

Figure 6 shows SEM micrographs of fracture surfaces of the bulk samples densified by CAPAD at different temperatures. At 1200 °C, there are many pores present in the material. As the temperature increases to 1300 °C the quantity and size of the pores decrease which corroborates the change in measured relative density from 91.2 to 97.5%. The morphology of microstructure of the samples densified at 1300 °C and above is consistent with well densified (low porosity) samples.

**Figure 7:**
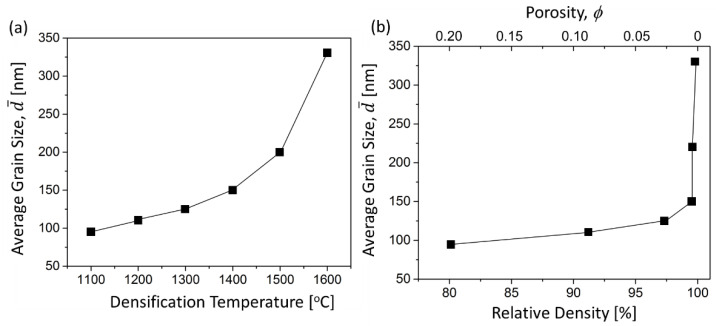
(**a**) Effect of CAPAD temperature on average grain size measured from the fracture surface of bulk body. (**b**) Grain size vs. relative density of bulk AlN.

Figure 7a shows the average grain size as a function of CAPAD temperature, using the same pressure (105 MPa) and hold time. As expected, the grain size of bulk AlN increases with the processing temperature. At the lowest temperature, 1100 °C, the grain size is below 100 nm and at 1600 °C the grains have grown to the sub-micron region >300 nm.

Figure 7b shows the relationship between relative density and grain size. There are two regions, densification dominant and grain growth dominant. Below 1300 °C, the density increased significantly while the change in grain size was not significant. The grain sizes obtained compare well with previous AlN nanopowder densification studies. For example, the combination of nanopowder and sintering additive resulted in grain size around 300 nm densified at pressure-less sintering 1500 °C [34]. Previous CAPAD studies on fine grain sized AlN reported 98% dense samples with final grain size around 200 nm [22,23]. By contrast, a sample produced here at 1400 °C has grain size ~150 nm and high density (99%). We believe this is the smallest grain size reported for AlN with porosity, Φ ≤ 0.01.

### 3.3. Densification of Nano-AlN Powder

**Figure 8:**
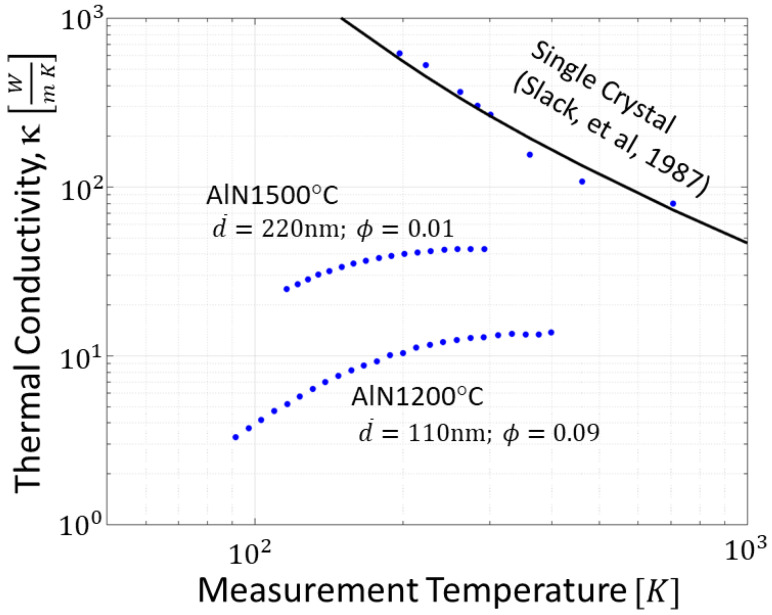
The temperature dependence of the thermal conductivity of polycrystalline AlN.

Figure 8 shows the temperature dependent thermal conductivity of nano-AlN samples densified at 1200 °C ($\bar{d}$ = 110 nm, 91% density) and 1500 °C ($\bar{d}$ = 220 nm, 99% density), with room temperature thermal conductivities of 13 W/(m·K) and 43 W/(m·K), respectively. These values are much lower than typical values for high quality single crystal AlN which can approach *κ_SC_* ≈ 300 W/(m·K) [9,10]. The dominant mechanism for the *κ* reductions seen in Figure 8 is the effect of grain boundary scattering [11,12] which is important whenever the average grain size $\bar{d}$ is comparable to or smaller than the intrinsic phonon mean free paths, Λ. For high-quality single crystal AlN, measurements [28] indicate that *κ_SC_* at room temperature is carried primarily by Λ ranging from ~100 nm to 10 μm. As a leading order estimate of the effects of grain boundary scattering, we make a gray (effective phonon) approximation of the phonon spectrum [35] and use Matthiessen’s rule to write:

(3) $$\kappa_{Dense}=\frac{\kappa_{SC}}{1+\Lambda_{gray}/\left(\alpha \;\bar{d}\right)}$$

where *κ_Dense_* is the expected thermal conductivity of a fully dense sample of the nanocrystalline material, α ≈ 0.86 accounts for grain boundary transmission [11], and we take $\Lambda_{gray}\approx 1\;\;\mu m$ as a representative gray mean free path (chosen as the geometric mean of the range 100 nm to 10 μm from Ref. [28]).

Porosity effects will further reduce the thermal conductivity, which we account for using a linear correction,

(4) $$\frac{\kappa_{Porous}}{\kappa_{Dense}}=1-\beta \varphi$$

where *φ* is the porosity and *β* is a scaling factor. In continuum heat conduction, the widely-used Maxwell-Garnett model [36] corresponds to $\beta =\frac{3}{2}$ for small *φ.* However, since the present samples have pore sizes smaller than the bulk Λ, ballistic effects will increase *β* and we use *β* ≈ 4 based on measurements of the porosity effect in nanocrystalline silicon [11].

Combining Equations (3) and (4) predicts *κ_Porous_* = 17 and 46 W/(m·K), respectively, for the 110 nm and 220 nm samples. These estimates are in reasonable agreement with the measured values of 13 and 43 W/(m·K) seen in Figure 8, thus supporting our interpretation that the thermal conductivity reduction is dominated by the effects of grain boundary scattering and porosity.

Despite the drastic reduction compared to single crystal values, the *κ* of nano-AlN bulk is still high compared to other monolithic nanocrystalline ceramics. For example, measurements nanocrystalline yttria stabilized zirconia has yield *κ* < 2 W/(m·K) [37] and the *κ* of strontium ferrite, SrFe_12_O_19_ in the cross-plane direction with grain sizes of ~300 nm is *κ* < 3 W/(m·K) [38]. Moreover, the value obtained are in the upper range or higher than conventional optical materials.

## 4. Summary

In summary, we have presented a synthesis route based on converting transition alumina (δ-Al_2_O_3_) with <40 nm grain size to AlN using a carbon free, ammonia gas reduction/nitridation process. This nano-AlN powder can be densified at significantly lower temperatures than high quality commercially available micrometer powder. The densified samples yield low porosity sample *φ* ≤ 0.01 with fine grain sizes. XRD result confirm that the bulk samples are primarily hexagonal AlN with some minor γ-ALON present as a second phase. The thermal conductivity of the dense nano-AlN samples is significantly reduced compared to single crystal AlN, since the average grain sizes are lower than the representative phonon mean free path ($\Lambda_{gray}\approx 1\;\;\mu m$). We believe that these are finest $\bar{d}$ reported in bulk dense AlN and is the first report of thermal conductivity for AlN with 110–220 nm grain size. The obtained *κ* proved the advantage of AlN over other conventional materials and the potential of AlN to be used for high-energy optical applications.
